# A High Aspect Ratio Bifurcated 128-Microchannel Microfluidic Device for Environmental Monitoring of Explosives

**DOI:** 10.3390/s18051568

**Published:** 2018-05-15

**Authors:** Paul T. Charles, Varun Wadhwa, Amara Kouyate, Kelly J. Mesa-Donado, Andre A. Adams, Jeffrey R. Deschamps, Anne W. Kusterbeck

**Affiliations:** Center for Bio/Molecular Science and Engineering, Code 6900, U.S. Naval Research Laboratory, Washington, DC 20375, USA; varun.wadhwa@nrl.navy.mil (V.W.); kouyateamara@gmail.com (A.K.); kdmesa@nrl.navy.mil (K.J.M.-D.); andre.adams@nrl.navy.mil (A.A.A.); jeffrey.deschampls@nrl.navy.mil (J.R.D.); anne.kusterbeck@nrl.navy.mil (A.W.K.)

**Keywords:** explosives, 2,4,6-trinitrotoluene (TNT), microfluidic, microchannel, immunoassay, fluorescence, antibody

## Abstract

Design and evolution of explosives monitoring and detection platforms to address the challenges of trace level chemical identification have led investigations into the use of intricately designed microfluidic devices. Microfluidic devices are unique tools that possess distinct characteristics that, when designed properly and configured with optical and fluidic components, can produce detection platforms with unmatched performance levels. Herein, we report the design, fabrication and integration of a bifurcated high aspect ratio microfluidic device containing 128 microchannels (40 mm × 40 μm × 250 μm; L × W × H) for explosives detection at trace levels. Aspect ratios measuring >6:1 support improved receptor-target molecule interactions, higher throughput and extremely low limits of detection (LOD). In addition to superior assay sensitivity, the bifurcated microfluidic device provides greater durability and versatility for substrate modification. Using the explosive 2,4,6-trinitrotoluene (TNT) as the model compound in a fluorescence-based displacement immunoassay, we report LODs for TNT at 10 parts-per-trillion (pptr) using a neutravidin-coated biotinylated anti-TNT microfluidic device. Solution to wall interactions were also simulated in COMSOL Multiphysics to understand fluid flow characteristics. Reynolds numbers were calculated to be 0.27–2.45 with a maximum pressure of 1.2 × 10^−2^ psi.

## 1. Introduction

Microfluidic devices and advances in microchip designs have spawned an evolution in new technologies targeted at improving sensor performance, clinical and environmental analysis and in-vitro diagnostics platforms [1,2,3,4,5]. Ranging in complexity, such devices offer a number of advantages: (1) low sample volume; (2) small form factor; (3) increased target-ligand interactions due to sub-micron scale dimensions; (4) high surface-to-volume ratio; (5) portability and (6) scalability. As scientists and engineers perfect fabrication techniques to manufacture these unique platforms (comprised of submillimeter dimensions), it has become more evident that fluid flow hydrodynamics and laminar flow properties can profoundly influence the molecular interactions at the micro- and nanoscale level [6]. Exploitation of these unique properties paired with advances in chemical surface modification methodologies has now enabled the next generation of sensor platforms that possess (1) a high degree of sensitivity and specificity for chemical or biological molecules; (2) versatility in all mediums; and (3) robustness for long-term usage. 

Microfluidic devices have proven their effectiveness in many disciplines of scientific research; proteomic analysis [7], macro-algae bloom investigations [8], biochemical mechanisms in 3D cell-based systems [9], paper-based microfluidic devices for biomarkers for disease and point of care diagnostics [1] and explosives detection [10,11]. Although traditional benchtop explosives detection systems and devices [12], and Singh’s method [13] continue to remain as the gold standard, microfluidic devices can in the near future provide a miniaturized, versatile, field portable platform capable of achieving the highest level of sensitivity.

The U.S. Department of Defense (DoD), environmental agencies and other global and national security agencies have a vested interest and continue to investigate new technologies especially in areas of explosives detection. Capillary-action driven microfluidic and paper-based devices combined with lab-on-a chip technologies [14] have demonstrated their use as novel technologies towards explosives analysis. Limits of detection for such devices have been reported at low nanogram levels**.** The micropad (μPAD), develop by Ueland and colleagues provided an interesting approach in using a wax printed paper-based microfluidic device for on-site screening of explosives in soil [15]. Based on fluorescence quenching paired with a 10 min extraction, step researchers were able to identify trace levels of eight explosives at quantities between 1–5 ng. Borowsky and colleagues assembled a planar microfluidic device (2.6 cm × 230 μm × 100 μm) with an integrated liquid-based chromatography system. This device was successful in the separation and detection of nitroaromatic and nitramine explosives [16]. Fabrication of the microfluidic device was accomplished through standard photolithography, sol-gel chemical synthesis along with electroosmotic flow (EOF) as a means of chemical elution and separation. Their results confirmed a correlation between peak resolution and voltage for both the parent explosives and their degradation products. Sample identification and peak resolution using this method proved very effective in delineating explosive compounds contained in complex mixtures.

Prior research within our group has focused on the development of technologies for the detection of explosives that provide; (1) a superior level of detection sensitivity; (2) flexibility to be miniaturized; (3) durability; (4) low cost to manufacture; and (5) high sample throughput. Investigations were conducted with a PMMA-modified 39-multichanneled microfluidic device for the detection of explosives [10,11,17,18] with targeted intentions for field applications. This report investigates a new design, fabrication, theoretical analysis and sensor integration of a 128-microchannel microfluidic device for explosives detection (specifically TNT) that offers the potential for enhanced sensor performance. Higher aspect ratios measured >6:1 support improved receptor-target molecule interactions, higher throughput and potentially lower limits of detection (LOD). COMSOL laminar fluid flow simulations, antibody receptor immobilization chemistries, fluorescence dye profiles at varied flow rates and limits of detection (LODs) for the explosive, TNT were investigated.

## 2. Materials and Methods

### 2.1. Microfluidic Device Fabrication

**Figure 1:**
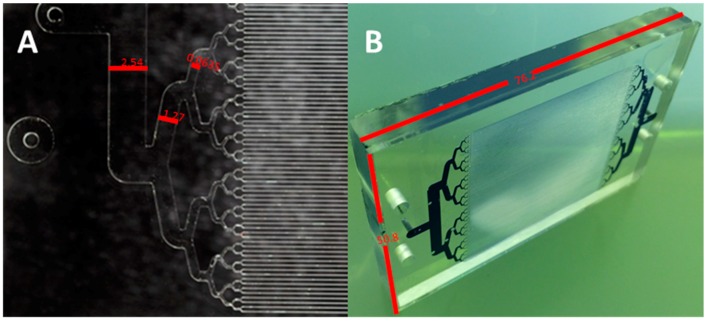
(**A**) Hot-embossed PMMA substrate with dimensions of 2.54 mm, 1.27 mm, and 0.635 mm from left to right. (**B**) Thermally annealed microfluidic device (L × W; 76.2 mm × 50.8 mm). Trypan blue dye added to the channels to delineate microchannels.

Prior research and design of immunosensors for explosives detection utilized a 39-microchanneled microfluidic device (paralleled; 25.4 mm × 250 μm × 500 μm) (L × W × D) [10,18]. High precision micromilling using a Haas Mini Mill (Oxnard, CA, USA) was used to fabricate a brass mold to produce the desired architectures. Using a 3-axis milling process the brass feed stock was machined to achieve a feature height of 500 μm using a combination of tapered and non-tapered flat end mill bits. The microstructures were generated by removing 0.02 mm layers with each pass. In a final step the features were profiled with a 0.12 mm diameter end mill-bit. Device profile measurements revealed an aspect ratio approximately 2:1. In an effort to further advance molecular interactions microfabrication of a 128-microchannel device with an aspect ratio >6:1 was performed. Microfluidic devices comprised of 128 microchannels (paralleled; 40 mm × 40 μm × 250 μm, L × W × D) (Figure 1) were fabricated via hot-embossing from a high precision micromilled brass mold tool. Precision micromilling of a brass feedstock (McMaster-Carr, Elmhurst, IL, USA; 0.635 × 30.5 × 30.5 cm^3^) using a Kern Precision, Inc. (Addison, IL, USA) micromill was performed initially to generate the desired microstructures into the brass feedstock. Milling bits (Bits and Bits Co., Silverton, OR, USA) were aligned using a 3-axis milling process in which a top layer of brass (500 μm) was removed to create a uniform top surface layer. Flat end mill bits (tapered and non-tapered) were used to create the desired microstructures. Instrument parameters included incremental passes at 2.5 μm with a uniform feed rate of 10.2 cm/min (*x*- and *y*-translations) plus a plunge rate of 2.5 cm/min (*z*-axis). Profilometry measurements were performed on the brass mold as a final characterization step to insure proper dimensions were achieved.

### 2.2. Hot Embossing of PMMA Substrate

Once the brass-mold was produced, a HEX02 hot-embossing machine (Jenoptik, GmbH, Jena, Germany) was used to hot-emboss the desired features into the surface of a poly (methyl methacrylate) (PMMA) substrate. PMMA, known for its inherent physical characteristics (durable, resistant to biofouling, and amenable to chemical surface modification) made its selection appropriate for this application. Substrates were hot-embossed from a brass molding tool that was 139.7 mm in diameter. Four 6.35 mm diameter through holes were used to align the embossing chamber and PMMA with the microarchitectures of the molding tool. Subsequent embossing steps were performed by placing the feature side up of the molding tool with the embossing chamber on top. A PMMA blank was then added to the assembly. Next, the polished brass substrate was placed polished side down on the assembly resulting in an embossed substrate that is optically transparent with minimal surface roughness (<0.025 mm). The aluminum embossing chamber was 6.35 mm thick and the PMMA feedstock was 9.52 mm thick which provided an excess of PMMA that resulted in a complete mold cavity fill during embossing. Once assembled, the PMMA-brass mold apparatus was then placed in an embossing press (in-house manufactured) between two temperature programmable heating platens with thermocouples to monitor the temperature during embossing. The lower platen in the press had a fixed *z*-axis and the upper platen was pneumatically actuated and capable of applying 40–120 psi of downward force to the stationary platen. Contact was initiated with the heating platens and the assembly while at room temperature then an applied force of 60 psi was applied. Temperatures of both the upper and lower platens were programmed to reach 170 °C maintained for a period of 45 min then cooled to 50 °C before the embossing press was opened. Embossed substrates were removed from the molding tool and trimmed to 10 × 10 cm^2^ using a band saw. Within the microarchitectures were four additional alignment markers which were used to drill through holes (3.0 mm dia.) with a drill press. Microfluidic conduits were also drilled for influent and effluent (1.0 mm dia.) passage.

### 2.3. Thermal Annealing of PMMA Substrate

Embossed PMMA substrates were sealed with PMMA coverslips (5.08 cm × 7.62 cm × 0.15 cm thick; prepared using an Epilog Laser Mini, Golden, CO, USA) of equal dimensions to produce the functional microfluidic devices. Inlet and outlet ports on the microfluidic device were drilled using a jeweler’s drill. Alignment holes to mount the microfluidic device were drilled using a 0.317 cm drill bit using a drill press. Each component was subsequently sonicated and cleaned in ultra-pure 18 megaohm (MΩ) H_2_O for 5 min to remove residual polymer debris caused during the drilling process. Each microfluidic device component (microchannel base and coverslip) was then UV-irradiated at 254 nm for 15 min at 100 Mw/cm^2^. Post-irradiation, each PMMA component was sonicated for 60 min at 25 °C to remove remaining debris. Thermal annealing of the embossed PMMA substrate and the coverslip was performed using a press with two heating platens. Each platen was set to 90 °C. A tri-solvent mixture (47.5% DMSO, 47.5% DI H_2_O, and 5% methanol) was applied between each substrate and pressure applied for 30 min to create a sealed microfluidic device. The microfluidic device was then cooled to 35 °C prior to being removed from the hot embossing press [18]. Quality control checks and leak tests were performed on each sealed microfluidic device by flowing 18 MΩ H_2_O through the microchannels at an initial flow rate of 0.1 mL/min with incremental steps to 1.0 mL/min.

### 2.4. COMSOL Simulation/Fluid Flow Simulations

**Figure 2:**
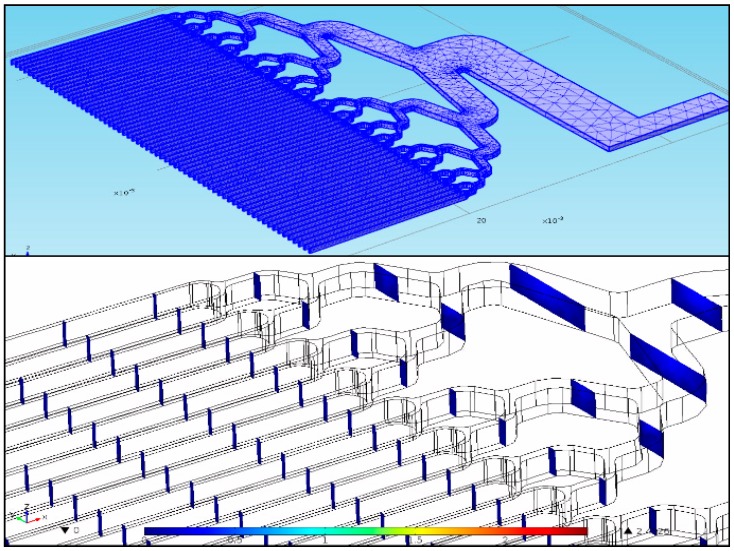
(**top**) An eighth of the proposed geometry with a free tetrahedral mesh and corner refinement. (**bottom**) Reynolds number simulation snapshot at a maximum value of 1.2 × 10^−2^ psi.

Laminar flow simulations at flow rates of 0.1 to 10 mL/min in steps of 0.5 mL/min were conducted with COMSOL Multiphysics (Burlington, MA, USA, Figure 2). In this simulation, 1/8 of the proposed geometry was imported into COMSOL (since the planes of symmetry served to diminish computing time and memory). A stationary solver and a free tetrahedral mesh element with corner refinement were used. The mesh elements sizes ranged from 8.28 × 10^−4^ m and 1.76 × 10^−4^ m, respectively. The boundary conditions at the inlet consisted of laminar inflow with an initial flow rate of 0.1 mL/min. The walls of the microfluidic were no slip boundaries.

### 2.5. Fluorescence Dye Profiles

Fluorescence calibration tests were performed with the fluorescent analog of TNT, cyanine 5-amine-TNB (Cy5-amine-TNB) to investigate fluorescence signal response and fluorescence peak profiles. In this series of experiments, concentrations of Cy5-amine-TNB (3.0 nM to 625 nM) were applied in triplicate to the microchannel microfluidic device and the fluorescence signal response was recorded using a fluorescence spectrofluorometer (FP2020-Plus, Jasco, Inc., Easton, MD, USA). Fluorescence signal responses were measured at flow rates of 1.0 mL/min and 2.0 mL/min with optical parameters for the spectrofluorometer preset for the Cy5-amine-TNB dye conjugate at excitation and emission wavelengths, 632 nm and 665 nm, respectively.

### 2.6. Synthesis and Purification of Cyanine 5-Amine-Trinitrobenzene (Cy5-Amine-TNB)

Cy5-amine-TNB (5.5 mg; 7.6 μmol) was dissolved in 500 μL of borate buffer saline (BBS), pH 9.6. TNBSA (10 μL; 1.7 μmol) was added directly to the Cy5-amine (5-fold excess) solution and allowed to react for 30 min at RT (covered in foil to shield from ambient light). Under basic conditions the cleavage of the sulfonate group will occur, resulting in product formation. Purification of the Cy5-amine-TNB product was achieved using an Infinity high-performance liquid chromatography (HPLC) system (Agilent, Santa Clara, CA, USA) equipped with an Agilent reverse-phase C18 semi-analytical column (Zorbax SB C18; 5 μm; 9.4 × 250 mm^2^). An acidic-methanol (10%): acidic-water (100%) gradient was applied with UV-Vis monitoring at 650 nm and 340 nm, respectively. Cy5-amine-TNB eluted at 19.8 min compared to unreacted Cy5-amine at 16.2 min. Product was collected, dried and stored in amber vials at −20 °C until further use.

### 2.7. Monoclonal Anti-TNT Immobilization (EDC Crosslinking)

Antibodies specific for TNT (1.0 mg/mL) (SDIx, Inc., Newark, DE, USA) were covalently immobilized onto the inner walls and bottom surface of the microchannel microfluidic device using standard EDC crosslinking chemistry [19]. Once immobilized, all antibody binding sites were saturated with Cy5-amine-TNB in excess (30 μM in PBS). Microfluidic devices were then wrapped in foil to protect from ambient light and stored at 4 °C until further use. Prior to immunoassay tests two microchannel volumes of PBS were applied to the microfluidic device to remove excess amounts of Cy5-amine-TNB. Constant monitoring of the fluorescence signal decrease was undertaken to establish a stable baseline before TNT samples were applied. Once the baseline was established TNT samples were applied incrementally.

### 2.8. Monoclonal Anti-TNT Immobilization (Neutravidin-Biotin Crosslinking)

Microfluidic devices containing biotinylated antibodies specific for TNT (1.0 mg/mL) coupled to a neutravidin-coated surface were prepared using a protocol previously described [11]. Pre-coating of the microchannels with a TEOS sol-gel solution was performed to create surface hydroxyls. These hydroxyl groups formed a “glass-like” layer required for covalent attachment of a thiol-terminated silane. A 4% solution of the thiol-terminated silane (3-mercaptopropyltrimethoxysilane) was infused into the device and allowed to incubate for 1 h at RT. Once the microfluidic device was treated with the silane, covalent attachment of the heterobifunctional crosslinker, GMBS was performed enabling a bridge for antibody conjugation. Utilizing the NHS-ester moiety of the GMBS crosslinker provided a reactive site for coupling the tetrameric protein, neutravidin. Neutravidin provides a strong non-covalent bond when coupled to a biotin-labeled protein or antibody. Biotin-labeled anti-TNT (1.0 mg/mL in PBS) was infused into the microfluidic device and incubated at 4 °C overnight. Upon conclusion of the antibody immobilization step, the fluorescent analog of TNT (Cy5-amine-TNB) was infused into the microchannels to saturate all antibody binding sites. Microfluidic devices not required immediately were saturated with a 1% solution of bovine serum albumin (BSA) prior to dye saturation and stored at 4 °C.

### 2.9. Explosives (TNT) Detection Assay

TNT displacement immunoassays were performed using the chemically modified anti-TNT microchannel microfluidic devices to determine its sensitivity and limits of detection (LOD) for the explosive compound, 2,4,6-trinitrotoluene (TNT). The fluorescence displacement immunoassay detection system consisted of the following components: (1) fluorescence spectrofluorometer-Model FP-2020 Plus—was used to measure the displaced fluorescence analog (Cy5-amine–TNB) from the binding pocket of the monoclonal antibody; (2) Rheodyne low pressure three-way-valve injector loop (100 μL) for sample introduction; and (3) two reciprocating pumps (Applied Biosystems, Inc., Foster, CA, USA) for continuous flow at a controlled flow rate (1.0 mL/min). As TNT is introduced into the continuous flow system, preferential binding occurs between the anti-TNT to TNT resulting in the displacement or release of Cy5-amine-TNB from the proteins binding site [20]. A resultant fluorescence signal (peak) over background is measured and correlated to the TNT concentration. TNT concentrations (0.001 ng/mL to 1000 ng/mL) were applied and the resultant fluorescence signal response were measured and quantified. Control samples of RDX (10 and 100 ng/mL) were also applied to the anti-TNT microchannel microfluidic device to measure antibody specificity and crossreactivity.

## 3. Results

A 128-microchanneled bifurcated microfluidic device with internal characteristics that possess a high surface-to-volume and high aspect ratio was investigated using COMSOL laminar flow simulations and immunodetection assays. Using a stationary solver and Navier-Stokes equation:

*ρ* (*u*·∇)*u* = ∇·[−*pI* + µ (∇*u* + (∇*u*)*^T^*)] + *F* (1)

where *ρ* is the fluid density, *u* is the velocity in (m/s), ∇ is the del operator, *p* is pressure (Pa), *I* gives the identity matrix, *F* is the internal force, and *T* is the deviatoric component of the total stress tensor that enabled a fundamental understanding of the microfluidic dynamics and aided in calculations to determine the Reynolds number and pressure loss across the microfluidic device. Equation (2) was used to calculate the Reynolds number [21] while also describing the ratio of inertial forces to viscous forces:

*Re* = *ρvD_H_*/μ (2)

where *D* is the diameter, *v* is the velocity, *ρ* is the density, and μ is the dynamic viscosity. The Hagen-Poiseuille equation, describes the pressure drop in a fluid flowing through a long cylindrical pipe:

Δ*P* = 8μ*LQ*/π*r*^4^ (3)

where Δ*P* is the pressure loss, *Q* is the volumetric flow rate, µ is the dynamic viscosity, *L* is the length of the pipe, and *r* is the radius [22]. In this study, assumptions of an incompressible laminar flow, isothermal conditions, negligible effect of gravity, and steady state condition were evident. Results from the COMSOL fluid flow simulations provided Reynolds numbers from 0.27–2.45 and a maximum pressure of 1.2 × 10^−2^ psi. Prior research using the 39-microchannel design demonstrated the significant influence on sensitivity of microfluidics from channel design wherein the aspect ratio was a dominant factor. Generally, aspect ratio scales directly with surface area to volume ratio (SA/V), as such solute interactions with wall bound capture elements increase, as well. Prior results obtained with the 39-microchanneled microfluidic device and results with the new 128-microchanneled design were consistent with initial assumptions by which a single-phase fluid flow through a relatively smooth microchannel surface is primarily under laminar flow with little to no resistance. This suggest the higher SA/V ratio measured in the 128-microchanneled device would be more efficient in the displacement-based immunoassay resulting in potentially lower limits of detection.

**Figure 3:**
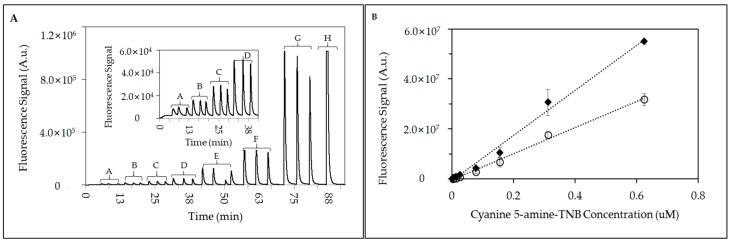
Fluorescence signal responses in microfluidic device. (**A**) Real-time fluorescence signal responses from triplicate injections of Cy5-Amine-TNB at 1.0 mL/min. Cy5-Amine-TNB concentrations applied: (A) 3.0 nM, (B) 6.5 nM, (C) 13.0 nM, (D) 25.0 nM, (E) 78.0 nM, (F) 156 nM, (G) 312 nM, and (H) 625 nM (single injection); (insect) Magnified fluorescence signal responses for Cy5-Amine-TNB at lower concentrations (A–D; 3.0 nM–25 nM). (**B**) Scatter plot of fluorescence peak area from triplicate injections of Cy5-amine-TNB through the 128 microchannel microfluidic device at (♦) 1.0 mL/min and (○) 2.0 mL/min.

Fluorescence dye profiles of Cy5-Amine-TNB at flow rates of 1.0 mL/min and 2.0 mL/min were recorded and plotted (Figure 3A,B). Results showed similar peak profiles in shape and amplitude as also seen in prior reports [10]. An immediate rise in the fluorescence signal over background upon excitation of the fluorescent complex (Cy5-amine-TNB) was observed. Minimal peak tailing was observed which traditionally indicates a diminishing concentration of the Cy5-amine-TNB as the fluorescent probe exits the flow cell chamber. Detectable concentrations of Cy5-amine-TNB were clearly measurable above the baseline from the lowest concentration applied 3.0 nM to 625 nM (Figure 3A). Figure 3A (inset) displays lower concentrations of applied Cy5-amine-TNB. Total fluorescence (peak area) observed at dye concentrations of 3.0 nM at flow rates of 1.0 and 2.0 mL/min were 334527 and 93349, respectively. Linear plots (Figure 3B) and calculated regression coefficients (R^2^) provided values of 0.994 and 0.991, respectively. 

**Figure 4:**
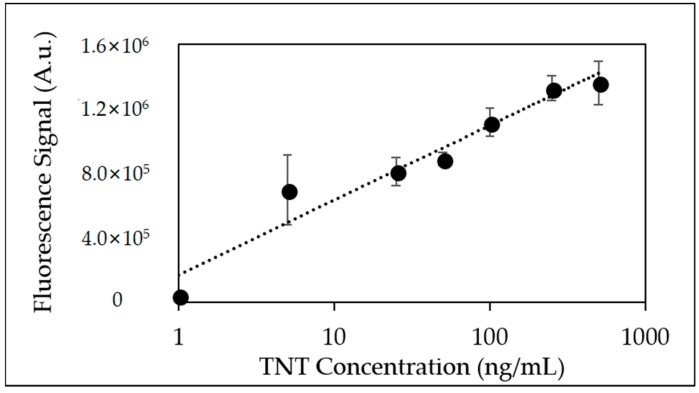
Linear plot of fluorescence peak area from triplicate injections of TNT (0.1 ng/mL–1000 ng/mL) using EDC immobilized anti-TNT coated microfluidic device.

Figure 4 depicts integrated fluorescence peak area from a TNT displacement immunoassay using the 128-microchannel microfluidic device where the anti-TNT was immobilized using traditional EDC chemistry. The displacement of the fluorescence analog was observed over four orders of magnitude with signal responses reported at the lowest TNT concentration injected (0.1 ng/mL). Observed fluorescence signal responses were measured at equal or higher levels based on previously reported linear plots [10]. Calculated R^2^ values over the entire concentration range was equal to 0.978 with percent standard deviation (%RSD) between 4–10% and signal-to-noise (S/N) ratios >7:1 (observed at TNT concentration 0.1 ng/mL). TNT displacement immunoassays were also performed with a microchanneled microfluidic device that employed a crosslinked neutravidin-biotinylated anti-TNT conjugated surface.

**Figure 5:**
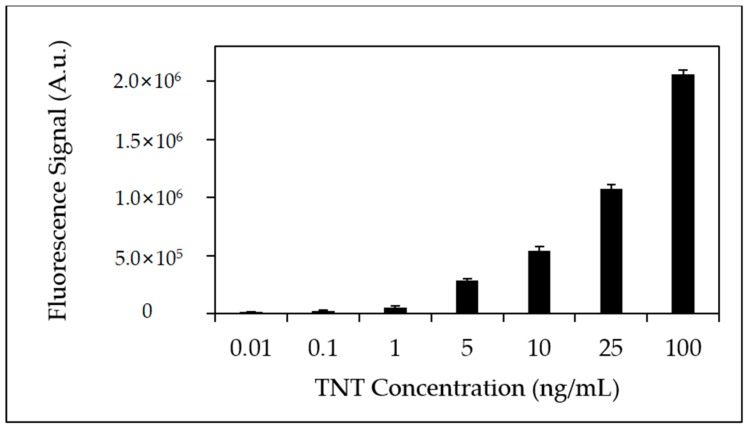
Bar plot of fluorescence peak area from triplicate injections of TNT (0.01 ng/mL–100 ng/mL) using Neutravidin-biotinylated anti-TNT coated microfluidic device.

Figure 5 depicts a bar plot of the fluorescence signal response from the TNT microchanneled microfluidic device with applied TNT concentrations (0.01–100 ng/mL). Fluorescence signal responses displayed an exponential rise with a calculated R^2^ equal to 0.999 with %RSD <5%.

**Figure 6:**
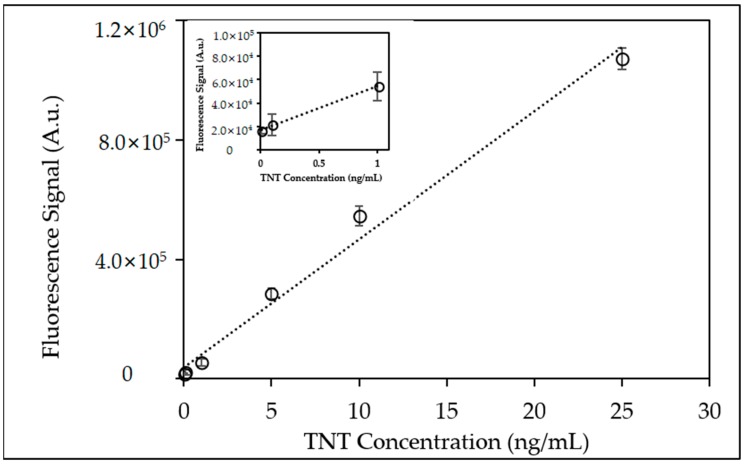
Scatter plot of fluorescence peak area from triplicate injections of TNT (0.01 ng/mL–25 ng/mL) using Neutravidin-biotinylated anti-TNT coated microfluidic device. (Inset) Scatter plot of fluorescence peak area at lowest injected TNT concentrations (0.01 ng/mL–1.0 ng/mL).

Figure 6 shows a scatter plot of the fluorescence signal responses shown in Figure 5 at the lower TNT concentration range (0.01–25 ng/mL). Results displayed a linear dynamic range that exists over four-orders of magnitude with a percent standard deviation (%RSD) <5%.

Using the neutravidin-biotinylated anti-TNT microfluidic device provided quantifiable detection limits at TNT concentrations as low as 0.01 ng/mL (10 pptr) (Figure 6; inset). Qualitative fluorescence signal responses for TNT at concentrations of 0.001 ng/mL (1.0 pptr) were observed but not did not provide quantifiable signal responses. 

**Figure 7:**
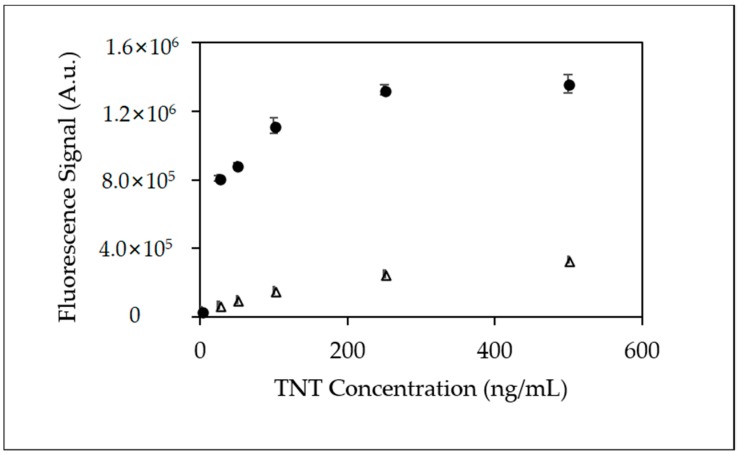
Scatter plot comparing fluorescence peak area from triplicate injections of TNT using Neutravidin-biotinylated anti-TNT coated microfluidic device (Δ) 39-microchannel and (•) 128-microchannel microfluidic device.

Figure 7 provides a comparison plot of fluorescence dose responses for both the 39-microchannel and the 128-microchannel device employing a TNT neutravidin-biotinylated anti-TNT coated surface. The dramatic differences in immunoassay response clearly demonstrate the combined benefits of the 128-microchanneled microfluidic device’s narrower channel design and its influence on the molecular interaction between anti-TNT and the TNT analyte. Fluorescence signal responses at many of the TNT concentrations were at least 2–3 fold higher.

**Figure 8:**
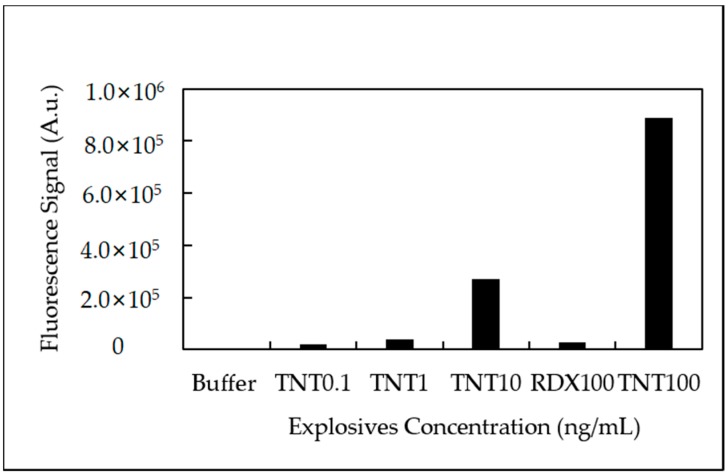
Antibody crossreactivity experiments. Injections of TNT (0.1, 1, 10 and 100 ng/mL) and RDX (100 ng/mL) to determine specificity of anti-TNT.

Crossreactivity measurements to demonstrate antibody specificity for TNT were also investigated using 1,3,5-trinitro-1,3,5-triazacyclohexane (RDX) as a control molecule (Figure 8).

RDX provided minimal to no fluorescence signal response when injected into the anti-TNT immobilized microfluidic device. The ratio of fluorescence signal response for TNT: RDX was approximately 34:1 (TNT 100 ng/mL) demonstrating the antibodies superior recognition capabilities for TNT.

## 4. Discussion and Conclusions

Demonstrated within this report is a 128-microchannel microfluidic device that combines intricate architectural designs and biomolecular specificity of an antibody specific for TNT for trace level TNT detection. Evolution of the displacement immunoassay format for TNT from porous membranes [23], fused-silica microcapillaries [24,25] to microchanneled microfluidic devices [20] signifies the importance and advantages of maintaining high surface area to volume ratios leading to increased antibody-antigen interactions. Characterization of the 128-microchanneled microfluidic device using COMSOL fluid flow simulations provided greater insight into the fluid dynamics and flow processes. Based on the calculated Reynolds numbers (*Re*) that ranged from 0.27–2.45 with a maximum pressure of 1.2 × 10^−2^ psi suggested minimal pressure resistance was observed resulting in a steady fluid flow through the microchannels. Although slightly higher *Re* values (e.g., 2.45) were measured in some microfluidic devices, these values provided no indication of surface roughness, increased pressures, turbulent flow or vortex formation.

Investigations also revealed the benefits of using a neutravidin-biotinylated anti-TNT conjugated microchannel surface for improved sensor response. TNT immunoassays provided quantitative trace level detection for TNT at concentrations of 0.01 to 10 parts-per-trillion (pptr). Fluorescence responses for TNT concentrations at 0.001 ng/mL (1.0 pptr) were recorded and measured but the signal-to-noise (S/N) was not deemed sufficient. In prior reports, the use of a single-chain variable fragment (scFv) biomolecule specific for TNT [11] on a neutravidin-coated surface displayed the benefits of the ligand complex and the residing surface. It is well known that neutravidin, which is a tetrameric protein complex, provides a number of advantages: (1) four binding sites for anti-TNT biomolecules; (2) stable, high dissociation constant (10^−15^ M) to biotin; and (3) a surface layer that prevents non-specific adsorption of proteins. Combining the chemical modifications to the surface, the flexibility of the biotin tether and the unique dimensions of the microchannels created a favorable environment to promote enhanced interactions between the anti-TNT and the TNT molecule. TNT immunoassay results comparing the 39- and 128-microchannel microfluidic devices clearly demonstrated the significance in the new 128-microchannel design and dimensions and its effect on immunoassay performance. Fluorescence signal responses were significantly higher with the 128-microchannel device indicating an enhanced effect on the molecular interaction between the anti-TNT and TNT analyte. Prior optimization of system parameters (e.g., antibody affinity, antibody density and fluid flow rate) enables the TNT molecule to diffuse towards the antibody binding site promoting successful displacement of the fluorescence TNT analog. Previous dissociation experiments conducted with a similar immunoassay displacement format have shown the significance and the influence of the antibodies affinity to TNT and the importance of the fluorescence analog’s dissociation rate in conjunction with the competitive binding for the TNT molecule [26] in order to achieve superior immunoassay sensitivity.

Antibody crossreactivity experiments revealed the importance of antibody specificity in the design of this TNT immunoassay detection system. Using an RDX control, TNT immunoassay results showed the clear difference in antibody recognition to TNT. This can be attributed to how the antigen is recognized in the antigen binding site and also immunogen development. TNT has a planar conformation where the nitro-groups are exposed out of plane on the aromatic ring. In contrast, the RDX molecule possesses a chair conformation with its nitro-groups in an axial position [18]. This distinction directly effects how the antibody recognition sites interact in the presence of TNT. In addition, antibody development is influenced by the immunogen which was a TNT derivative with a keyhole limpet hemocyanin (KLH) [26]. 

In our efforts to design miniature, portable and robust detection platforms these new experimental findings suggest the progression from the 39-microchannel device to the new 128-microchannel microfluidic device has the potential for even higher signal responses and lower limits of detection for use in environmental remediation efforts, hazardous chemical run-off detection and port and harbor security.
